# Influence of ethephon and soil treatments on the essential oil composition of sweet fennel and its biological activities

**DOI:** 10.1038/s41598-024-82204-1

**Published:** 2024-12-23

**Authors:** Suzan Adib Mina, Mokhtar Mohamed Bishr, Hoda Mazyoun Hassan, Soad Mohamed Abdel-Khalik

**Affiliations:** 1 Department of Pharmacognosy, Faculty of Pharmacy, Helwan University, POB 1179, Cairo, Egypt; 2 pharmaceuticals and medicinal plants (Mepaco), Enshas El Raml, Sharkyah Egypt

**Keywords:** *Foeniculum vulgare*, Cytotoxic, Cultivation, Antimicrobial, Essential oil, Estragole, Cancer, Plant sciences, Environmental sciences

## Abstract

The manipulation of cultivation conditions in addition to environmental changes is a key factor affecting the growth, secondary metabolites production, and the biological activity of the plant. Our study aims to evaluate the impact of foliar spray of ethephon, water stress, organic and inorganic fertilizers on the cytotoxic and antimicrobial activities of the fruit oil of *Foeniculum vulgare*, Miller, var. *dulce* and its vegetative growth and oil composition. Four different soil treatments were implemented. Gas chromatography coupled with mass spectrometry GC/MS was applied to identify the volatile compounds. Evaluation of both the cytotoxic and the antimicrobial activities of the fruit oil were assessed using the viability assay and the well diffusion method respectively. Variations in the vegetative growth factors and the essential oil (EO) composition were recorded. About a 94.7% increase in the total oil yield was detected in condition 3 compared to control. The oil sample obtained under condition 1 showed the strongest antimicrobial activity against most tested strains and a significant cytotoxic effect against prostate carcinoma (PC-3) cells. Facing the global problem of water deficit, our success in producing a good quality fennel crop and its EO content upon application of drought stress and will be very beneficial at both the economic and the pharmaceutical levels.

**Supplementary Information:**

The online version contains supplementary material available at 10.1038/s41598-024-82204-1.

## Introduction

Sweet fennel (*F. vulgare* var. *dulce*) is a well-known aromatic medicinal plant, which is used, in traditional medicine as a spice and as a substrate for different industrial purposes^1^,^2^. The essential oil of fennel is mainly concentrated in the fruits (mericarps) and provides the unique aroma and taste of sweet fennel. Anethole, fenchone, and estragole are among the main oil constituents. The oil with lower fenchone and higher trans-anethole ratios makes the aroma of fennel fruits sweeter and more delicate^3^,^4^. According to The Committee on Herbal Medicinal Products (HMPC), exposure to estragole, a naturally occurring genotoxic carcinogen^5^,^6^ should be kept as low as practically achievable.

Environmental stress and climate changes impact agricultural production and food supply^7^,^8^. They are the primary causes of crop losses and the reduction of average yields for most major crops by more than 50%^9^,^10^. Both the severity and the duration of the applied drought stress can affect the composition of the EO^11^,^12^ Previously reported data shows that drought stress leads to reduced plant heights, leaf area indices, and head yields^13^,^14^ while it leads to increased EO yield in the range of 2.21–2.42%^15^. Growth regulators (e.g. Ethephon) are recommended as they are known to affect physiological and biochemical processes or even gene regulation. The foliar spray technique promotes more rapid absorption of nutrients directly applied to the demand location in the leaves^16^,^17^.

Since the compositions of the essential oils are very much influenced by both intrinsic and extrinsic factors several previous attempts to investigate the possibilities of organic farming for the production of fennel in Egypt as reported in^18^,^19^ compared the difference between natural and synthetic sources of nitrogen added during fennel cultivation on its oil yield. The present study reveals the modification in both the cytotoxic and the antimicrobial activities of *F. vulgare* Miller var. *dulce* as a consequence of both the variation in vegetative growth factors and the volatile oil composition in response to altering the agricultural conditions. We aim to achieve a controlled cultivation condition for *Foeniculum vulgare* Miller var. *dulce* which can give the highest productivity and quality of oil needed in the pharmaceutical industry with the best biological activity.

## Materials and methods

### Plant material

*Foeniculum vulgare*, Miller, var. *dulce*, family Apiaceae, fruits were obtained from Mepaco Company. Egypt. The plant was botanically identified and authenticated by Dr Abdul Halim Abdul Majali Mohammed, Head of Flora Research and Plant Classification Department, Agricultural Research Centre. A voucher specimen number (2Fvu2/2017) was deposited in the herbarium of Pharmacognosy Department, Faculty of Pharmacy, Helwan University.

### Materials used

#### Materials used for cultivation

Ethephon 48% (Ripex 48%) StarChem Company, Super Phosphate (12.5% P_2_O_5_) Abo Zabal Company for fertilizers. Ammonium sulfate (20.6% N) Kian for international commerce, Egypt. Chicken Fertilizer, Chicken Farm, Sharkyah, Egypt.

#### Materials for the cytotoxic activity

Dimethyl sulfoxide (DMSO), crystal violet, and trypan blue dye were purchased from Sigma (St. Louis, Mo., USA). Growth medium (Fetal Bovine serum, Dulbecco’s modified Eagle’s medium DMEM, RPMI-1640, HEPES buffer solution, 1% L-glutamine, 50 µg/ml gentamycin and 0.25% Trypsin-EDTA were purchased from Lonza). The reference drug (doxorubicin) was purchased from Sigma (St. Louis, Mo., USA).

#### Materials for the antimicrobial activity

The inoculum suspension was prepared from colonies grown overnight on an agar plate and inoculated into Mueller-Hinton broth (fungi using malt broth). Reference drugs Ketoconazole and Gentamycin from Sigma/ Aldrich, (USA).

### The experimental design and treatment before and after sowing

For the application of four different soil treatments compared to control (Fig. S11, Table 1), the land to be cultivated was divided into five plots of 15 m^2^ area. Each plot was divided into seven rows. Fennel fruits were planted at the rate of five fruits per hole at a distance of 40 cm. Germination was practically completed after 12 days from sowing for all conditions except control in which germination was completed after one month.

**Table 1:** Summary of the four applied soil treatments before and after sowing fennel fruits.

Treatments Condition	Before sowing	After 45 days	After 75 days	Irrigation schedule
Control (1^rst^ plot)	No treatment	No treatment	No treatment	Every 15 days
Condition 1 (2nd plot)	Chicken manure (1Kg/m^2^)	Ethephon (240 ppm)	Ethephon (240 ppm)	Every 15 days
Condition 2 (3rd plot)	Ca (H_2_PO_4_)_2_ (250 g)	(NH_4_)_2_SO_4_ (720 g) + Ethephon (240 ppm)	(NH_4_)_2_SO_4_ (720 g) + Ethephon (240 ppm)	Every 15 days
Condition **3** (4th plot)	Chicken manure (1Kg/m^2^)	Ethephon (240 ppm)	Ethephon (240 ppm)	First 4 months every 15 days remaining irrigation every 45 days
Condition **4** (5th plot)	Ca (H_2_PO_4_)_2_ (250 g)	(NH_4_)_2_SO_4_ (720 g) + Ethephon (240 ppm )	(NH_4_)_2_SO_4_ (720 g) + Ethephon (240 ppm)	First 4 months every 15 days Remaining irrigation every 45 days

### Essential oil extraction for yield calculation

100 g of fresh fruits from each plot was extracted using a conventional Clevenger-type hydro distillation apparatus. In this type of extraction, the plant material was immersed in one-liter water in a round flask connected to a Clevenger-type apparatus. Heating was conducted using a heat mantle for three hours. The extraction temperature was adjusted to 100 °C. The oil was transported along with water vapor to a separate part of the apparatus. The resulting oils were collected, dried over anhydrous sodium sulphate, and stored in a refrigerator until analyzed. Percentage yields were expressed as volume of essential oil per dried material weight (%V/W) according to^20^.)

### Headspace GC sampling

Fresh fruits, obtained from each condition, were separated from the rest of the plant and subjected to headspace extraction followed by GC-MS analysis. The extraction procedures were performed at incubation temperature (130 °C), incubation time (120 min), syringe temperature (140 °C), and agitator speed (250 rpm).

### Gas chromatography/mass spectrometry

GC/MS analysis was performed on a GC/MS system (Shimadzu-2010 Ultra GC/MS) with software (Class 5000). Gas chromatograph equipped with a column Tr-5MS (5% Phenyl polyphenylene siloxane), column (DB-5, 30 m × 0.25 mm i.d × 0.25 μm film thickness). Carrier gas: Heluim with flow rate 1 ml/min; Detector temp. Flame ionization detector (FID): 230 °C; Injector temp.: 210 °C; split ratio; 1: 10; Oven temp. Program: initial temp.; 40 °C (2 min) increasing to 210 °C (at 5 °C/min) − 210 °C (5 min). The capillary column was directly coupled with mass spectrometer HP 5973 (Agilent). EI-MS were recorded at 70 ev. The analysis has been done at the Quality Control Department, Arab CO. for Pharmaceutical and Medicinal Plants (MEPACO), Cairo, Egypt.

### Identification of volatile constituents

Volatile components peaks were identified by their Kovats retention index (KI) relative to n-alkanes (C6-C20), mass spectrum matching to NIST, WILEY library database.

### Cytotoxicity evaluation using viability assay

For cytotoxicity assay, the cells were seeded in a 96-well plate at a cell concentration of 1 × 10^4^ cells per well in 100 µl of growth medium. A serial two-fold dilutions of the five tested samples were added. All experiments were carried out in triplicate. Control cells were incubated without a test sample. After incubation viable cells yield were determined by a colorimetric method using a Microplate reader (SunRise, TECAN, Inc, USA) at a test wavelength of 490 nm. The survival curve of Hepatocellular carcinoma cells (HepG-2) and prostate carcinoma cells (PC-3) was plotted after treatment with the specified oil sample (Fig. S1- S5) and the 50% inhibitory concentration (IC_50_) was determined against each type of cell. Using Graph pad Prism software (San Diego, CA. USA)^21,22^. The cancer cell lines were obtained from VACSERA Tissue Culture Unit and doxorubicin was used as a reference standard.

### Evaluation of antimicrobial activity by well diffusion method

The antimicrobial activity was tested by well diffusion method using 100 µl of suspension of the tested microorganisms containing 2 × 10^8^ CFU/ml for bacteria, and 1.5 × 10^6^ CFU/ml for fungi. A sterile swab was immersed in the suspension to inoculate Mueller-Hinton agar plates (fungi using malt agar plates). The five samples were dissolved in dimethyl sulfoxide (DMSO) with different concentrations (10, 5, 2.5 mg/ml) and applied to the wells in equal volumes. The plates were left for 30 min at room temperature to allow the diffusion of the oils, and then they were incubated at 37 °C for 24 h for bacteria and at 28 °C for 48 h for fungi. Controls using DMSO were adequately done. All experiments were carried out in triplicate. The diameters of the inhibition zones (mm) were measured^23^. Microbial strains of *Cryptococcus neoformas* (RCMB 0049001), *Staphylococcus aureus* (RCMB 010010), *Bacillus subtilis* (RCMB 015 (1) NRRL B-543), *Salmonella typhimurium* (RCMB 006 (1) ATCC 14028), *Escherichia coli* (RCMB 010052) ATCC 25,955 were obtained from Regional Center For Mycology and Biotechnology (RCMB), Al-Azhar University. Reference drugs Ketoconazole and Gentamycin from Sigma/ Aldrich, (USA).

### Statistical analysis

All tests were conducted in triplicates and values are recorded as mean and standard deviations. Parametric analyses were assumed. Differences between means were determined using one-way analysis of variance (ANOVA) and Tukey’s test as a post hok test for multiple comparisons. All tests were done using GraphPad Prism 5.

## Results

### The growth and the EO yield

Thirteen vegetative growth parameters were measured (Table 2) to monitor the effect of the four applied cultivation conditions. It was noted that most of the measured parameters were significantly ameliorated, especially the fruit oil yield. The fruit oil yield was significantly affected with the application of foliar spraying of ethephon and drought stress. About 94.7% and 91.18% increase in the total oil yield was detected respectively in conditions 3 and 4 in comparison to control. It is worth mentioning that also, great similarity in measurements was recorded between condition 1 and condition 2. In both conditions, a normal irrigation schedule and foliar spray of ethephon were applied.

**Table 2:** Different parameters measured under the influence of four different cultivation treatments.

Condition Parameters	Control	C1	C2	C3	C4
Root length (Cm)	22.67 ± 11	50 ± 3^***^	51 ± 3^***^	33 ± 2	33 ± 2
Root diameter (Cm)	1.800 ± 1	2.8 ± 0.78	2.9 ± 0.75	1.6 ± 0.32	1.6 ± 0.32
Stem height (Cm)	114.7 ± 26.8	138 ± 4	136 ± 5	107 ± 3	102 ± 3
Stem diameter (Cm)	1.800 ± 0.2	3.0 ± 0.57	3.1 ± 0.57	2.1 ± 0.54	2.1 ± 0.54
No. of umbels/plant	9.333 ± 4.1	22 ± 2^***^	23 ± 2^***^	14 ± 1	15 ± 1
No. of rays/umbel	22.3 ± 1.53	19.7 ± 0.577	21.0 ± 1.73	30.7 ± 2.08^***^	30.3 ± 2.52^**^
No. of fruits/plant	204	440	483	462	495
Wt. of fruits /plant (g)	0.91	3.79	4.22	3.90	4.24
Umbel diameter (cm)	13.33 ± 1.7	9 ± 0.8^***^	10 ± 0.1^**^	7 ± 0.6^***^	7 ± 0.9^***^
Whole plant fresh wt. (g)	291 ± 2.08	378 ± 2.2^***^	368 ± 1.9^***^	297 ± 2.5^*^	281 ± 2.1^**^
whole plant dry wt (g)	120 ± 32.3	129 ± 2.1	124 ± 2.6	103 ± 1.6	100 ± 1.4
Wt of 100 fruits (g)	0.448 ± 0.09	0.862 ± 0.31	0.875 ± 0.33	0.846 ± 0.25	0.857 ± 0.26
Yield of fruit oil (% V/W)	1.7%	2.20%	2.16%	3.31%	3.25%

C1-4 = Conditions 1–4, No = number, Wt = weigh.

*compared to control: ^***^: *p* < 0.001, ^**^ : *p* < 0.01, ^*^: *p* < 0.05, Data expressed as the mean value ± S.D.

### The composition of fruit oil

The effect of the four applied cultivation conditions on the fruit oil composition of *F. vulgare* var. *dulce* was recorded in (Table 3, Fig. S6- S10). Fruit oil samples obtained under cultivation conditions (1–3) were dominated by hydrocarbons with the major class being monoterpenes while the oxygenated compounds dominated the control oil sample. The study showed differences in the oil constituents of fruit, especially in trans-anethole, estragole, fenchone, limonene, and α-pinene. Manipulation of soil treatment of *F. vulgare* Miller, var. *dulce* contributed greatly to the amelioration of the quality of the obtained oil based on comparison to a control plot in which more than half the oil composition was formed of estragole (53.27%) in addition to the presence of the lowest limonene percentage. The application of inorganic fertilizers (Ca (H_2_PO_4_)_2_ and NH_4_SO_4_) in conjunction with foliar spray of ethephon and the implementation of a regular irrigation schedule led to the accumulation of trans-anethole as the major constituent (34.91%) in the oil obtained under condition 2. Limonene appeared with the highest percentage in condition 3 which represented nearly up to half the composition of the oil sample. α-Pinene followed limonene in abundance, as it appeared with its highest percentage in condition 1 (14.74%). Sesquiterpenes formation seems to be independent of the type of added fertilizers.

**Table 3:** The effect of four soil treatments and irrigation regime on oil composition of *Foeniculum vulgare* Miller var. *Dulce* fruits (in fruiting stage).

^*^Rt	^**^KI	Components	control	C1	C2	C3	C4
			% component				
5.178	806	2,3-Butanediol	0.5	–	–	–	–
8.027	923	*α* -Thujene	–	–	0.36	–	0.27
8.322	933	*α* **-Pinene**	**--**	**14.74**	**12.37**	**2.08**	**12.14**
8.67	952	Camphene	–	1.88	1.27	0.29	1.46
9.531	980	2- β -Pinene	–	1.14	1.14	–	0.74
10.054	991	β -Myrcene	–	6.09	4.98	1.86	4.61
10.417	1007	1-Phellandrene	–	1.98	1.5	–	1.14
10.797	1012	α -Terpinene	–	0.48	0.59	–	0.4
11.425	1031	Limonene	9.82	26.56	26.37	48.59	23.23
11.52	1031.8	Pantolactone	0.55	–	–	–	–
11.58	1040	Cis-Ocimene	–	4.34	3.25	2.34	2.66
11.989	1047	1 H-Pyrrole-2-carboxaldehyde	1.06	–	–	–	–
12.151	1059	γ -Terpinene	–	1.28	2.65	0.29	1.14
13.138	1087	Fenchone	–	6.68	4.68	2.29	7.01
13.41	1094	iso amyl-2-methyl butyrate	–	–	0.13	–	–
13.568	1104	Isoamylisovalerate	–	0.05	0.08	–	–
13.845	1115	D-Fenchyl alcohol	–	–	–	–	0.12
14.258	1118	Maltol	0.58	–	----	–	–
14.266	1132	Neoalloocimene	–	0.3	0.21	–	0.25
14.780	1139	Camphor	–	0.12	0.09	–	0.13
15.786	1179	4-Terpinenol	–	–	0.14	–	0.23
16.474	1200	Estragole	**53.27**	**3.09**	**3.65**	**4.43**	**16.22**
17.049	1231	β -Fenchyl acetate	0.88	–	0.06	0.64	0.25
17.45	1236	α -Fenchyl acetate	0.49	0.09	0.09	0.84	0.1
17.99	1250	Trimethyl-tetrahydronaphthalene	–	0.21	–	–	0.24
19.333	1283	trans-anethole	**31.49**	**29.59**	**34.91**	**30.83**	**26.9**
21.3	1384	p-Acetonylanisole	0.42	–	–	–	–
21.452	1391	α -Copaene	–	0.14	0.17	–	–
24.2	1480	Germacrene-D	–	0.25	–	–	–
28.968	1685	Apiol	–	–	–	–	–
34.535	1874	Ethanone, 2,2-dimethoxy-1,2-diphenyl	0.55	–	–	–	–
		Essential oil %v/w	1.7	2.2	2.16	3.31	3.25
		Total identified cpds	11	19	21	11	20
		% identification	99.61	99.01	98.69	94.48	99.24
		Hydrocarbons%	9.82	59.39	54.86	55.45	48.28
		Monoterpene%	9.82	58.79	54.69	55.45	48.04
		Sesquiterpene %	–	0.39	0.17	–	–
		Trimethyl-tetrahydronaphthalene	–	0.21	–	–	0.24
		Oxygenated cpds%	89.79	39.62	43.83	39.03	50.96
		alcohol	1.08	-----	0.14	-----	0.35
		Ether	85.18	32.68	38.56	35.26	43.12
		Esters	1.37	0.14	0.36	1.48	0.35
		Ketones	0.55	6.8	4.77	2.29	7.14
		Aldehyde	1.06	------	------	-------	------
		Lactones	0.55	---	---	---	---

^*^Rt = retention time, ^**^KI = Kovats index, C1-4 = Conditions 1–4.

### Cytotoxic activity

All tested fruit oil samples showed significant anticancer activities against the two tested cell lines (Table 4). According to the National Cancer Institute (USA), vegetable crude extracts with IC_50_ values less than 30 µg/ml in tumor cell line assays are considered promising cytotoxic drugs^24^. It was noted that the control oil sample afforded the lowest IC_50_ values for HepG-2 cells (1.39 ± 0.0157 µg/ml) and (PC-3) cells (2.89 ± 0.0623 µg/ml).

**Table 4:** IC_**50**_ values of *F. Vulgare* Miller var. *Dulce* fruit oil samples compared to doxorubicin against two human cell carcinoma:

Cell type	Mean IC_50_ ± S.D					
	Doxorubicin	Control	C1	C2	C3	C4
***HepG2***	0.343 ± 0.0873	1.39 ± 0.0157	7.56 ± 0.402^***^, ^*aaa*^	9.50 ± 0.898^***, *aaa*^	15.1 ± 2.21^***^, ^*aaa*^	8.28 ± 1.18^***, *aaa*^
***PC-3***	3.18 ± 0.770	2.89 ± 0.0623	5.01 ± 0.837	11.0 ± 1.29^***, *aaa*^	8.43 ± 0.663^***, aaa^	5.31 ± 0.950 ^*a*^

*Data expressed as the mean value of* 50% inhibitory concentration (IC_50_ in µg/ml) *± S.D*, C1-4 = Conditions 1–4.

* Level of significance compared to doxorubicin: ***: *p* < 0.001, ** : *p* < 0.01, * *p* < 0.05.

^*a*^*Level of significance compared to (control): aaa*: *p* < 0.001, aa: *p* < 0.01, ^a^ : *p* < 0.05.

### Antimicrobial activity

**Fig. 1:**
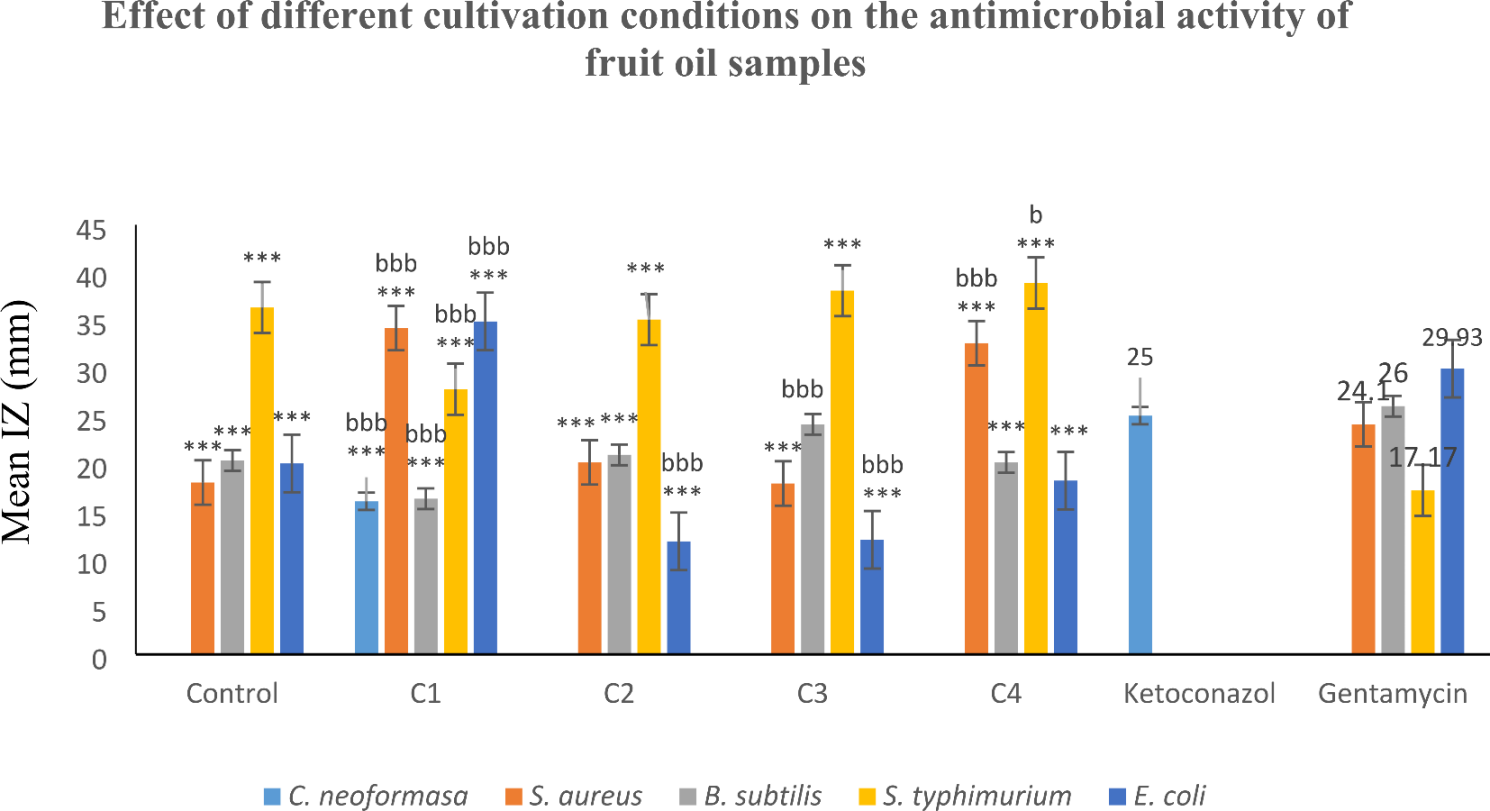
The effect of different soil treatments on the antimicrobial activity of the essential oil of the fruit of *F. vulgare* var. *dulce*.

Nowadays, the increasing resistance of pathogens to antibiotics presents a major threat to public health^25^. It was recently reported that monoterpene and sesquiterpene hydrocarbons and their oxygenated derivatives exhibit potential antimicrobial activities^26^. Our results showed that the gram-positive and gram-negative strains of bacteria had different sensitivities to different EO samples of fennel fruits. Among these bacteria, *S. typhimurium* is the most susceptible organism to all tested oil samples compared to the reference drug. It is worth mentioning that the sensitivity of *S. typhimurium* to different oil samples was more than doubled for all oil samples (in comparison to the reference drug) except for the oil sample obtained under condition 1 which was the most significantly effective against both *E. coli* and *S. aureus* in addition to being the only sample showing antifungal activity in comparison to control oil sample (Fig. 1; Table 5). Both oil samples obtained under conditions 1 and 4 showed significant inhibitory activity against *S. aureus* (in comparison to the reference drug and control oil sample). *B. subtilis* was more sensitive to oil samples from condition 3 (in comparison to the control oil sample).

**Table 5:** The effect of different cultivation treatments on the antimicrobial effect of the fruit oil samples of *F. vulgare*, Miller, *var*. *Dulce* plant against different strains of gram + ve, gram –ve, and fungi.

Tested sample and control drug	Fungi	Gram +ve		Gram – ve	
	C. neoformasa	S. aureus	B. subtilis	S. typhimurium	E. coli
**Control**	NA	18.0 ± 1^***^	20.3 ± 0.58^***^	**36.33 ± 1.53** ^***^	20.00 ± 1^***^
**C1**	**16.03 ± 0.15** ^***,bbb^	**34.17 ± 0.76** ^***,bbb^	16.3 ± 0.26^*** ,bbb^	27. 77 ± 0.68^*** ,bbb^	**34. 87 ± 0.32** ^***,bbb^
**C2**	NA	20.1 ± 1.15^***^	20.9 ± 0.81^***^	35.07 ± 0.90^***^	11.83 ± 0.76^***,bbb^
**C3**	NA	17.9 ± 0.81^***^	24.1 ± 0.81^bbb^	**38.10 ± 0.36** ^***^	12.00 ± 1.00^***,bbb^
**C4**	NA	**32.6 ± 0.51** ^***, bbb^	20.1 ± 0.81^***^	**38.90 ± 0.75** ^***,b^	18.20 ± 0.72^***^
**Ketoconazol**	25 ± 0.006	–	–	–	–
**Gentamycin**	–	24.1 ± 0.06	26 ± 0.153	17.17 ± 0.06	29.93 ± 0.153

*Data expressed as the mean value of inhibition zone (mm) ± S.D.*,* statistical significant at P≤*,* compared to either ketoconazol and gentamycin.*

* compared to reference drug:^***^: *p* < 0.001, ^**^ : *p* < 0.01, ^*^ : *p* < 0.05.

*b compared to (control)*: :^*bbb*^: *p* < 0.001, ^bb^ : *p* < 0.01, ^b^ : *p* < 0.05.

NA: No activity.

## Discussion

### The effect of different cultivation conditions on the growth and the EO yield

It was evident that a normal irrigation schedule in the presence of spraying with ethephon irrespective of the type of fertilizers used led to a better growth rate for all plant organs^27^,^28^,^29^. The study also showed the importance of combining foliar spraying with ethephon and applying water stress (drought) during irrigation, which may be explained by the potentiation of ethylene production which promotes fruit maturation in which the oils are mainly produced^30^,^31^. Our results comply with the reported data of increased oil yield under drought stress^32^,^15^. Also, adding chicken manure (organic fertilizer) in condition 3 favors increased fruit oil content^33^. It was evident that irrespective of the type of treatments added to the soil during the cultivation of fennel fruits, drought stress was the main factor controlling the increase in the total fruit oil yield, as by comparison of conditions 1/3 and 2/4 were the same soil additives were applied and the only difference was the irrigation regiment. About 94.7% and 91.18% increases in the total oil yield were detected respectively in conditions 3 and 4^15^.

### The effect of different cultivation conditions on the constituents of fruit oil samples

All applied cultivation conditions in our study favored the methylerythritol phosphate biosynthetic pathways over the mevalonate pathway. These led to the accumulation of higher percentages of monoterpenes (48.04–58.79%) over sesquiterpenes (0.17–0.39%). In contrast, the control plot was dominated by oxygenated compounds representing 89.79% with the major class being ether (85.18%), including 31.49% for anethole, and more than half of the oil composition was formed of estragole (53.27%). Although the oil sample obtained under condition 1 and condition 2 show the highest anethole/estragole ratio (9.6) among all tested samples, still, consider the oil sample obtained from condition 3 to be better as it has an appreciable anethole/estragole ratio (≃7) with the added value of possessing the highest total oil yield and the largest limonene content (nearly double the amount detected in the oil samples obtained under condition 1 and condition 2). In addition to a lower fenchone content which imparts the bitterness to the oil taste, thus leading to a better oil quality from the pharmaceutical point of view.

### The effect of different cultivation conditions on the cytotoxic activity of fruit oil

As per our results of GC/MS analysis of the tested samples, the presence of the highest content of trans-anethole, limonene, Myrcene, alpha, and beta-pinene in oil samples obtained under conditions 1, 2, 3 may rationalize the significant anticancer activity seen for these samples^34^,^35^,^36^,^37^; through synergistic effect^38^. The lowest IC_50_ values for HepG-2 cells and (PC-3) cells were recorded for the control oil sample which may be explained by the presence of estragole reaching up to (53.27%), known for its cytotoxic effect. Under the restriction of the European Pharmacopoeia (2nd edition) for the content of estragole which must be kept below 10%^5^,^39^ in addition to many studies evaluating the cytotoxic, genotoxic, and apoptotic activities of estragole; this sample cannot be considered as the best option.

### The effect of different cultivation conditions on antimicrobial activity

From the results in (Fig. 1; Table 5), The oil sample obtained under condition 1 possesses the highest monoterpenes hydrocarbons content (58.79%), which may play a role in rendering the oil more hydrophobic and thus showing a stronger inhibitory effect against different bacterial strains^40^,^41^. The oil sample obtained under condition 1 was also the richest in α-pinene, β-myrcene, and cis-ocimene. Due to the complexity of the composition of the oil, it is hard to identify a single component as being responsible for the biological activity of the oil it is rather due to a synergistic effect. It can be concluded that applied cultivation condition 1 favors the production of the oil showing the highest activities with the best oil quality.

## Conclusion

The study showed that the application of different cultivation conditions led to the improvement of most of the measured parameters of vegetative growth compared to the control sample in addition to promising biological activities (cytotoxic and antimicrobial effect). The highest fruit oil yield and quality were obtained by concomitant application of chicken manure, spraying with ethephon, and a water deficit irrigation regime (condition 3). The obtained essential oil contains the highest limonene content and a high percentage of trans-anethole. Moreover, it shows the lowest percentages of both estragole and fenchone which imparts the bitterness to the oil taste. The oil obtained under condition 3 will be very beneficial at both the economic and the pharmaceutical levels. Our success in producing good quality fennel crops with the best EO content and more irrigation control helps us face the global problem of water deficit.

## Electronic supplementary material

Below is the link to the electronic supplementary material.

Supplementary Material 1

## Data Availability

Data is provided within the manuscript or supplementary information files.
