# Supplementary material for: Influence of ethephon and soil treatments on the essential oil composition of sweet fennel and its biological activities
Source: Sci Rep. 2024 Dec 23;14:30609. doi: 10.1038/s41598-024-82204-1 (PMC11666597; doi:10.1038/s41598-024-82204-1)

**Online resources**

**Influence of ethephon and soil treatments on the essential oil composition of sweet fennel and its biological activities.**

B

A

**Fig. S1** The dose-response curve of fruits oil sample of *F. vulgare*, Miller, var. *dulce* plants cultivated as **(control)**, A= Hepatocellular carcinoma (HepG-2), B= prostate carcinoma (PC-3)

B

A

**Fig. S2** The dose-response curve of fruit oil sample of *F. vulgare*, Miller, var. *dulce* plants obtained from condition 1, A= Hepatocellular carcinoma (HepG-2), B= prostate carcinoma (PC-3)

A

B

**Fig. S3** The dose-response curve of fruit oil sample of *F. vulgare*, Miller, var. *dulce* obtained from condition 2, A= Hepatocellular carcinoma (HepG-2), B= prostate carcinoma (PC-3)

B

A

**Fig. S4** The dose-response curve of fruit oil sample of *F. vulgare*, Miller, var. *dulce* plants obtained from condition 3, A= Hepatocellular carcinoma (HepG-2), B= prostate carcinoma (PC-3)

B

A

**Fig. S5** The dose-response curve of fruit oil sample of *F. vulgare*, Miller, var. *dulce* obtained from condition 4, A= Hepatocellular carcinoma (HepG-2), B= prostate carcinoma (PC-3)

**Fig. S6** GC/MS chromatogram of *F. vulgare* Miller, var. *dulce* fruit oil obtained from the control
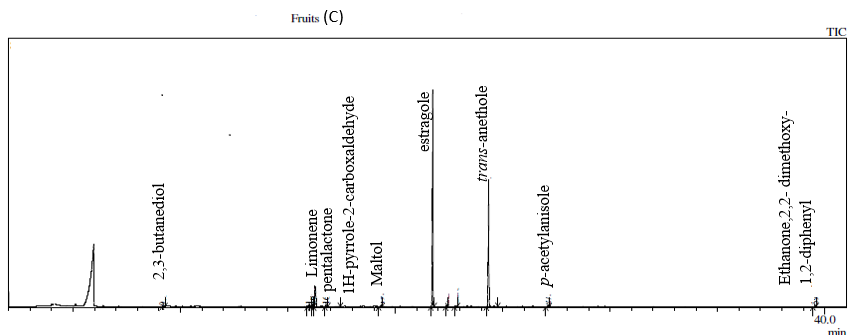


**Fig. S7** GC/MS chromatogram of *F. vulgare* Miller, var. *dulce* fruit oil from condition 1


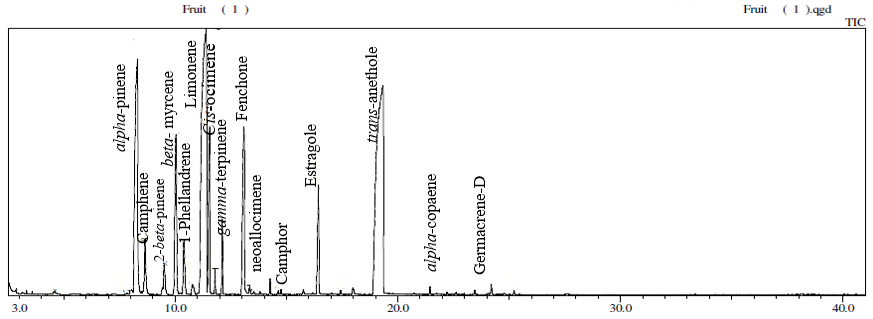


**Fig. S8** GC/MS chromatogram of *F. vulgare* Miller, var. *dulce* fruit oil obtained from condition 2


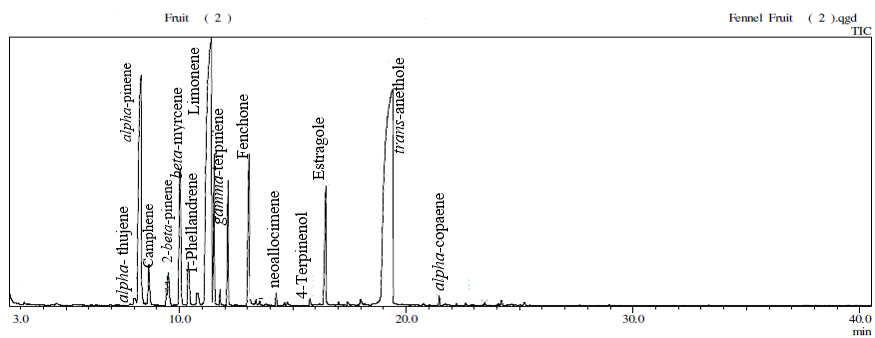


**Fig. S9** GC/MS chromatogram of *F. vulgare* Miller, var. *dulce* fruit oil obtained from condition 3


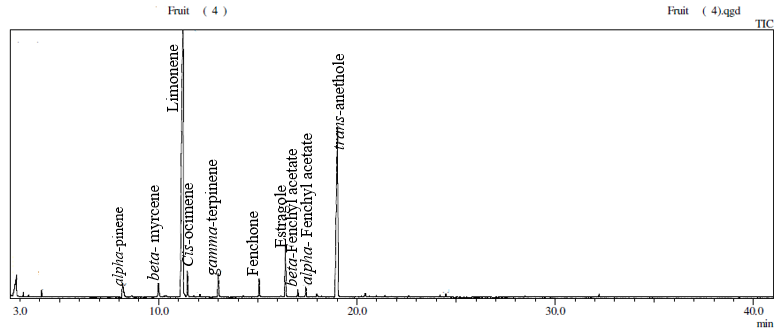


**Fig. S10** GC/MS chromatogram of *F. vulgare* Miller, var. *dulce* fruit oil obtained from condition 4


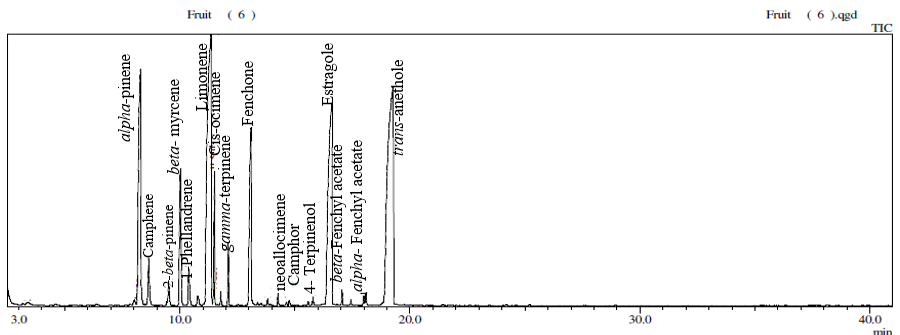


**Fig. S11** Schematic diagram of the four applied soil treatments before and after sowing fennel fruits


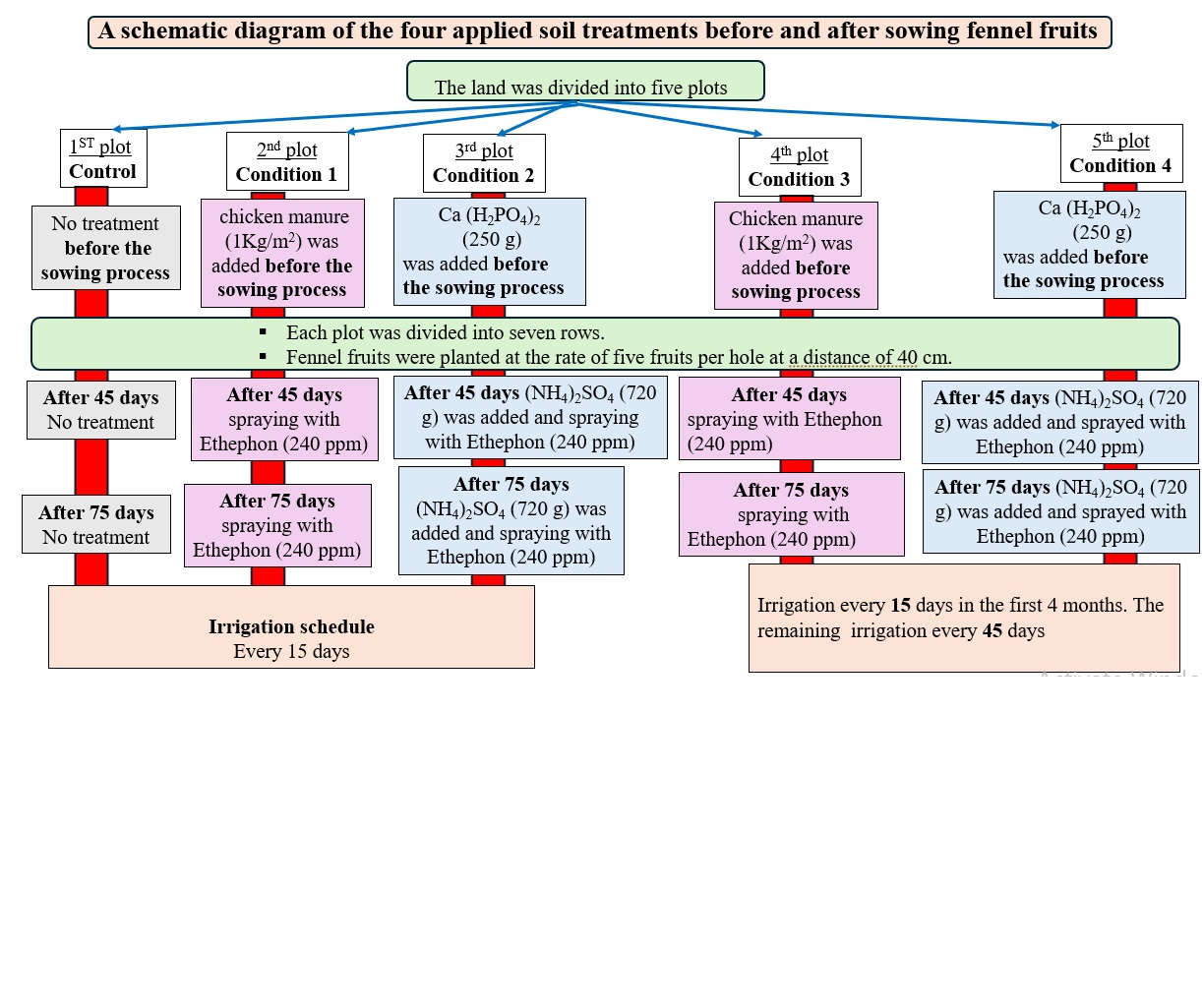


**Fig. S12** The antimicrobial effect of the fruit oil samples of *F. vulgare*, Miller, *var. dulce* plant against different strains of Gram +ve, Gram –ve, and fungi


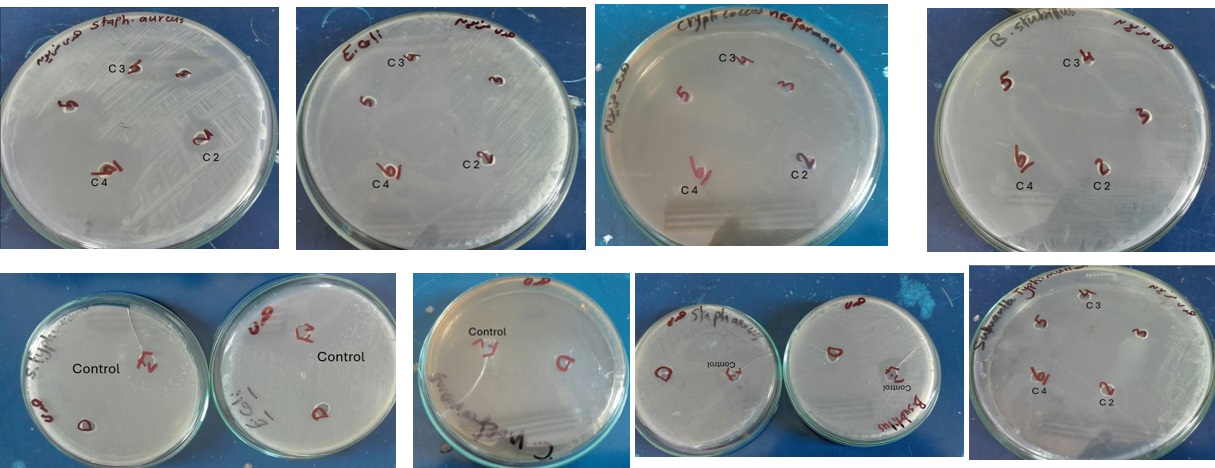

Supplement: Supplementary file 1 — Supplementary Material 1 [file 41598_2024_82204_MOESM1_ESM.docx]
